# Design, Fabrication, and Performance Characterization of LTCC-Based Capacitive Accelerometers

**DOI:** 10.3390/mi9030120

**Published:** 2018-03-09

**Authors:** Huan Liu, Runiu Fang, Min Miao, Yichuan Zhang, Yingzhan Yan, Xiaoping Tang, Huixiang Lu, Yufeng Jin

**Affiliations:** 1 National Key Laboratory of Science and Technology on Micro/Nano Fabrication, Peking University, Beijing 100871, China; liuhuan169@pku.edu.cn (H.L.); fangruniu@pku.edu.cn (R.F.); yfjin@pku.edu.cn (Y.J.); 2 Institute of Information Microsystem, Beijing Information Science and Technology University, Beijing 100085, China; zhangyichuan@rails.cn; 3 China Electronics Technology Group Corporation No. 54 Research Institute, Hebei 050081, China; yyz712@gmail.com (Y.Y.); xptang54@gmail.com (X.T.); luhuixiang54@gmail.com (H.L.); 4 Shenzhen Graduate School of Peking University, Shenzhen 518055, China

**Keywords:** low-temperature co-fired ceramic (LTCC), capacitive accelerometer, wireless, process optimization, performance characterization

## Abstract

In this paper, two versions of capacitive accelerometers based on low-temperature co-fired ceramic (LTCC) technology are developed, different with respect to the detection technique, as well as the mechanical structure. Fabrication of the key structure, a heavy proof mass with thin beams embedded in a large cavity, which is extremely difficult for the conventional LTCC process, is successfully completed by the optimized process. The LC resonant accelerometer, using coupling resonance frequency sensing which is first applied to LTCC accelerometer and may facilitate application in harsh environments, demonstrates a sensitivity of 375 KHz/g over the full scale range 1 g, with nonlinearity less than 6%, and the telemetry distance is 5 mm. The differential capacitive accelerometer adopting differential capacitive sensing presents a larger full scale range 10 g and lower nonlinearity less than 1%, and the sensitivity is 30.27 mV/g.

## 1. Introduction

Low-temperature co-fired ceramic (LTCC) technology, which was initially applied for RF applications, is one of the integration techniques for microelectronic systems. Due to the ability to embed integrated passive devices into substrates and good electrical properties, such as low dielectric loss and high-speed transmission thanks to the usage of low dielectric ceramic and highly-conductive Ag/Pd/Au conductors, LTCC technology is widely used in the field of microwave circuits and highly-reliable electronic military components [1,2]. LTCC technology enables the fabrication of 3D structures by micromachining perforated features into individual green tape and then laminating and sintering the multilayer stack to form the compact integrated substrate/interposer. The merit is soon exploited by various applications, including biomedical devices, electrochemical devices, microfluidic devices, pressure sensors, and temperature sensors [3,4,5,6,7]. In the field of micro-accelerometers, silicon-based accelerometers have been widely used in inertial measurement, aerial navigation, and gravity gradient measurement [8,9,10]. LTCC-based accelerometers utilizing different sensing principles were also reported. Neubert et al. [11] reported the first LTCC accelerometer, which uses piezoresistors and measures the voltage gap in the bridge circuit to determine the acceleration. Subsequently, Jurkow et al. [12] proposed an LTCC accelerometer utilizing the piezoelectric effect. A patented PZT film was applied to the surface of LTCC membrane as the acceleration-sensing component. Moreover, a triaxial LTCC accelerometer using piezoresistors is proposed as the follow-up [13]. An early exploration of a LTCC capacitive accelerometer was conducted in [14], which mainly focused on simulation, and a fabrication process based on sacrificial material was conceived.

Due to the hermeticity, chemical inactivity, and high-temperature stability of the LTCC material, one major advantage of LTCC-based sensors over their silicon counterparts is the resistance to harsh environments [15], which facilitates the application of LTCC-based sensors in harsh environments where silicon-based sensors cannot be deployed. Additionally, LTCC-based sensors can be easily integrated in multi-component modules (MCMs) which usually use LTCC substrates as a platform to achieve a compact-sized system [3]. Compared to LTCC accelerometers based on piezoresistive and piezoelectric principles, which may introduce materials incompatible with LTCC and instability at high temperature, the capacitive accelerometer is more suitable for high-temperature applications. In this paper, the LTCC-based capacitive accelerometers are designed, fabricated, and characterized. The conventional LTCC process is unable to fabricate the key structure for the capacitive accelerometer, namely, a heavy proof mass with thin beams embedded in a large cavity, because the structure would collapse during co-firing. Thus, an optimized LTCC process flow is developed to solve the problem. Based on the acceleration-sensitive structure, two signal processing methods are applied to capacitive accelerometers: one is telemetry of the resonance frequency between the sensor and the readout unit by inductive coupling, and the other is translating the differential capacitive input into a voltage output using a commercial readout chip. The performances of accelerometers are confirmed by experiments, which demonstrate good wireless acceleration-input transmission for the LC resonant accelerometer, and stable performances for the differential capacitive accelerometer.

## 2. Structure Design and Process Optimization

**Figure 1:**
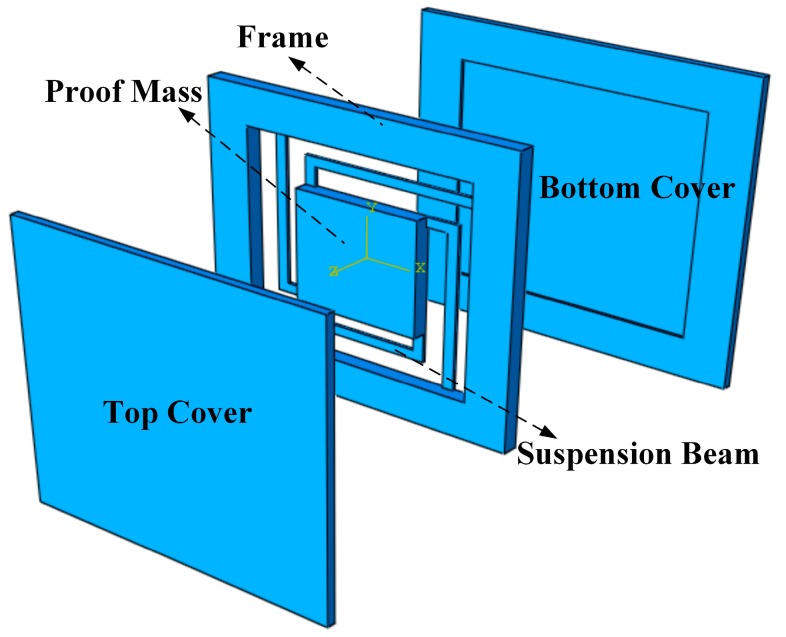
Schematic of the LTCC accelerometer.

The structure of the LTCC accelerometer is shown in Figure 1, which consists of three parts. In the middle part, the proof mass is suspended by four symmetrical beams. By screen-printing metal on the proof mass, it acts as a movable electrode, thereby forming a variable capacitor with top or bottom electrodes. Movement of the proof mass due to an out-of-plane acceleration causes changes in capacitance. 

**Figure 2:**
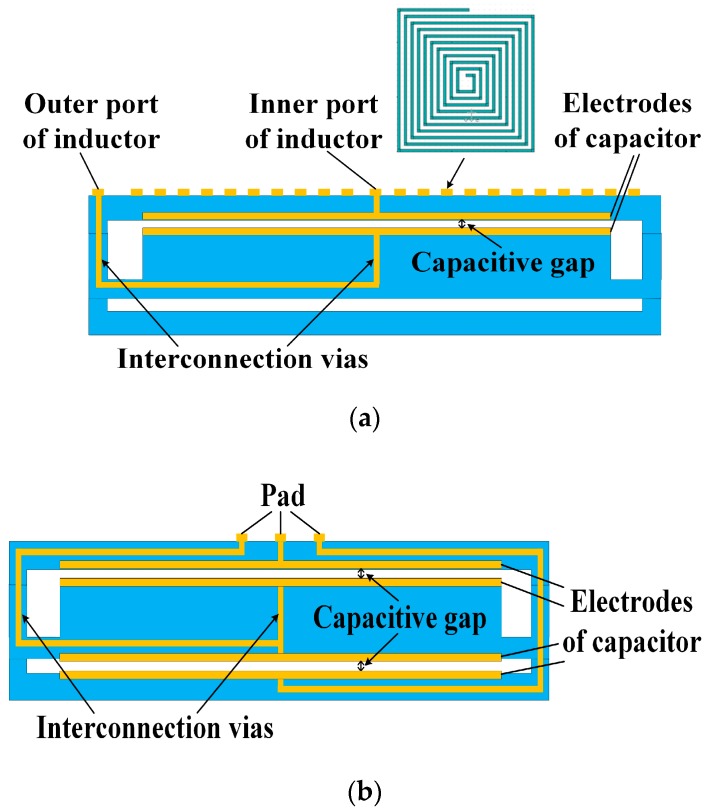
Profile of the accelerometers: (**a**) LC resonant accelerometer; and (**b**) differential capacitive accelerometer.

**Figure 3:**
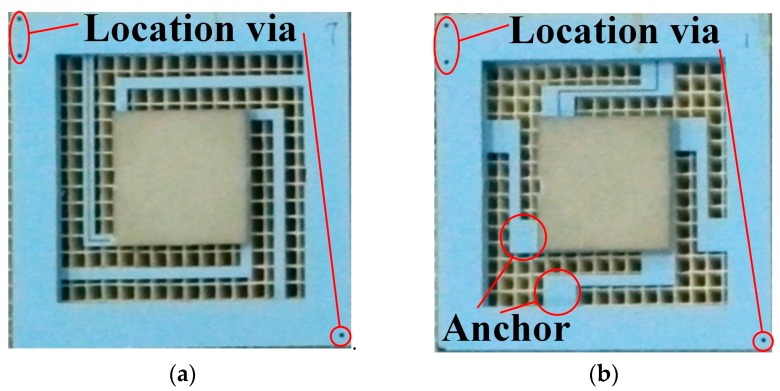
Beam-mass structure: (**a**) L-shapedd beams used in the LC resonant accelerometer; and (**b**) Z-shapedd beams used in the differential capacitive accelerometer.

The seismic middle part is sandwiched between top and bottom covers, which protect the sensitive structure and form a cavity for the proof mass to vibrate. In this paper, we applied two signal processing methods to the accelerometer structure. One method is the coupling resonant frequency sensing, which embeds a variable capacitor and an inductor in the accelerometer, and measures the resonance frequency of the LC circuit by a remote reader coil. The other method is differential capacitive sensing, which embeds a pair of differential capacitors into the accelerometer, and then translates differential capacitive input into voltage output using a commercial readout chip. Figure 2 shows the profile of the LTCC accelerometers. For the LC resonant accelerometer, a variable plate capacitor is formed between the top cover and the proof mass by screen printing electrodes on them. A spiral inductor is printed on the surface of the top cover, and wired to the capacitor’s electrodes with vertical interconnection vias and horizontal interconnections. The differential capacitive accelerometer has a similar profile, and will be discussed in detail in Section 3.2. The beam-mass structure has a significant effect on the performance of the accelerometer, such as the measuring range and sensitivity, therefore, two types of beams, L-shapedd beams and Z-shapedd beams, are designed and fabricated as shown in Figure 3, and the location vias are designed for precise alignment of different parts in fabrication. Since coupling resonance frequency sensing is more sensitive to noise, the L-shapedd beam, which is easier to deform, is used to guarantee a high sensitivity. Additionally, differential capacitive sensing is more stable, and the Z-shaped beam is used to achieve a large measuring range. 

Table 1 lists the designed physical dimensions of the accelerometers. The overall dimension of the accelerometer is 30 mm × 30 mm × 2.3 mm.

Numerical simulations were performed to obtain the mechanical behavior of the beam-mass structure using FEM (finite element method) software ANSYS (ANSYS Inc., Canonsburg, PA, USA). The material properties are referred to in [16]. Due to its longer effective beams, the L-shaped beam-mass structure demonstrates much higher sensitivity than that of the Z-shaped beam-mass structure, which is 2.99 μm/g compared with 0.321 μm/g. However, the trade-off between sensitivity and bandwidth results in a lower resonance frequency for the L-shaped beam-mass structure. The first resonance frequency of the two beam-mass structure is 291 Hz and 885 Hz, corresponding to a vibration of the proof-mass in the Z direction. The next two modes following the first mode are torsional vibration around the two diagonal lines of the proof-mass, respectively. The simulation results are listed in Table 2. It is noted that the accelerometers are rotationally symmetrical about the center of the proof-mass, and the angle of rotational symmetry is 90 degrees, so the second and third resonance frequencies are the same due to equal stiffness and moments around the *X*-axis and *Y*-axis for these two modes.

**Figure 4:**
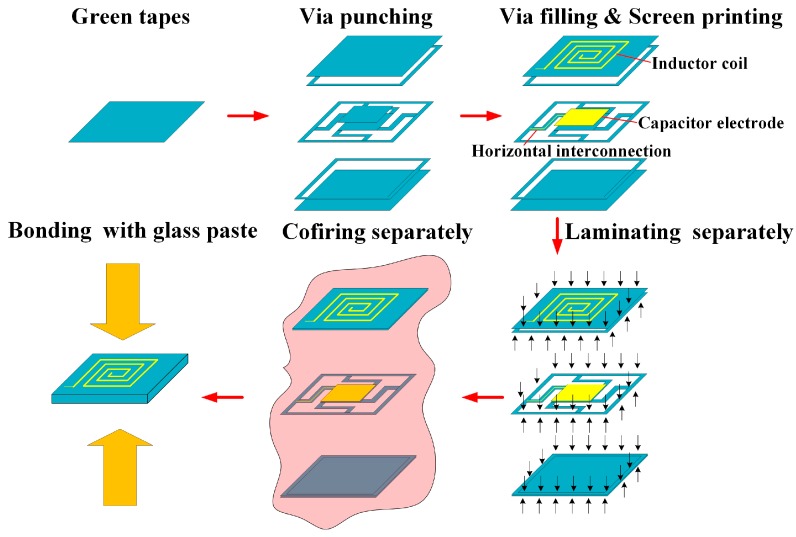
Optimized process flow for LTCC accelerometers.

The accelerometers were fabricated with LTCC technology, but the traditional LTCC process has difficulties in fabricating the high-quality large cavity and beam-mass structures of accelerometers. To solve this problem, an optimized process, as shown in Figure 4, is proposed.

Green tapes, which consist of alumina ceramic-filled glass systems mixed with an organic vehicle, are a basic material of LTCC technology. They are available from commercial suppliers at different thickness, and Dupont 951PT green tapes with 100 μm thickness were adopted in the fabrication. Depending on the thickness, the accelerometer needs 23 layers of green tape in total, of which four layers are for the top cover, 15 layers are for the middle frame, and four layers are for the bottom cover.

After preparing green tapes, the process moved to the via punching step to fabricate signal interconnection vias, location vias, and cavities. The programmable punching machine is controlled by a document which records the patterns of the green tapes.

Then the interconnection vias were filled with metal paste (Dupont Ag) using screen printing techniques, and the inductor, capacitor electrodes, and horizontal interconnection lines were also screen-printed.

The major difference between the proposed process and traditional LTCC process are subsequent steps. For the traditional LTCC process, after the previous steps, all green tapes will be laminated and co-fired together. However, the features of the accelerometer, particularly the large cavity with dimensions of 22 mm × 22 mm × 1.7 mm embedded in the structure and enormous difference in the mass of the beams and proof mass (the mass ratio of the proof mass to beams is 45 for L-shaped beams and 30 for Z-shaped beams), imposed a great challenge to fabrication because unfired green tapes were in a relatively soft state, the movable thin beams could not support the heavy proof mass structure, and they would collapse in the cavity.

In most cases, sacrificial layers that are easy to burn out, such as graphite powder-based paste, can be applied to solve this problem [17,18]. This method is typically used to fabricate cavities and channels free of deformation. The sacrificial layer supports the three-dimensional structure up to the burnout temperature during co-firing and, when the structure is stiff enough, it is burned out into gas and escapes from the intrinsic pores in green tapes, which is followed by densification and elimination of the pores of the LTCC tapes. Control of the burnout characteristics of the sacrificial layer is critical for this method. If the sacrificial layer starts to burn out before the tapes become stiff, the embedded structure will sag or even collapse. If the burnout of the sacrificial layer is not complete after the tapes’ densification, the gas generated afterward will swell the tapes. Sagging and swelling problems also have a negative effect on the interconnections located on the surface of tapes. For the designed accelerometer, fabrication of the cavity is a challenge, and the existence of the suspension proof-mass makes it more difficult because neither of the two covers of the accelerometer could touch the proof-mass and the space is only 100 μm. Therefore, the next steps were optimized for the accelerometer, where the three parts of the accelerometer were laminated and co-fired separately and then bonded together with glass paste.

In step 4, the green tapes of each part were stacked, and these three parts were laminated separately in a laminating machine which adopts isostatic pressing in heated water. The process setting is isostatic hydraulic pressure of 20 MPa in 70 °C water for ten minutes. The parts were vacuum sealed in a plastic bag to prevent the water from coming into contact with them.

Then the three parts were co-fired separately. The temperature profile of co-firing is as follows: 20–400 °C for 5 h to volatize the organic particles; 400–600 °C for 6 h for structure formation, and the green tapes started to harden around 500 °C; then, 600–900 °C for 5 h for complete densification; and 900–20 °C for 3 h for cooling down.

The final step is bonding with glass paste. First, the three parts were aligned precisely with the help of a computer aided vision system, images of location vias on the surface of LTCC tapes to be aligned were captured by a CCD (charge-coupled device) camera (Sony, Tokyo, Japan), and alignment is accomplished by adjusting the images until they coincide. Then the stack was sintered at 600 °C, and a good bonding strength can be achieved because both glass and LTCC are isotropic materials. 

**Figure 5:**
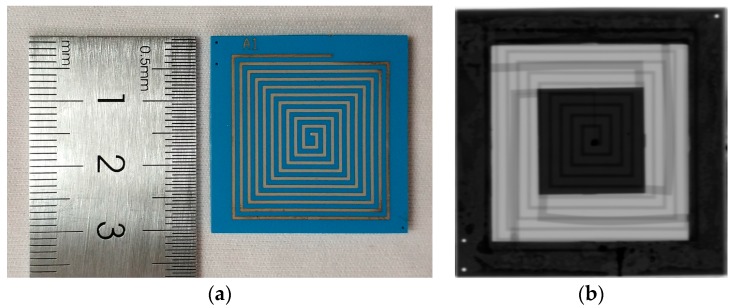
Fabricated LTCC accelerometers: (**a**) optical image; and (**b**) X-ray inspection image.

The optimized LTCC process flow is very useful to fabricate movable structures in LTCC substrates, where cavities can be avoided during co-firing and, thus, more control on movable structures during fabrication can be obtained. With the process optimization, the LTCC accelerometers were fabricated successfully, as shown in Figure 5, and the X-ray inspection image proved the structural integrity.

## 3. Signal Processing Methods

In this section, the two different signal processing methods applied on the designed accelerometers are discussed in detail, which are coupling resonance frequency sensing and differential capacitive sensing. By using the coupling resonance frequency sensing, the accelerometer can be easily deployed in harsh environments for its separated sensing circuits and reader antenna, but the involved signal processing is complicated, while the differential capacitive sensing is more stable because of its fully-developed interface circuit.

### 3.1. Coupling Resonance Frequency Sensing

**Figure 6:**
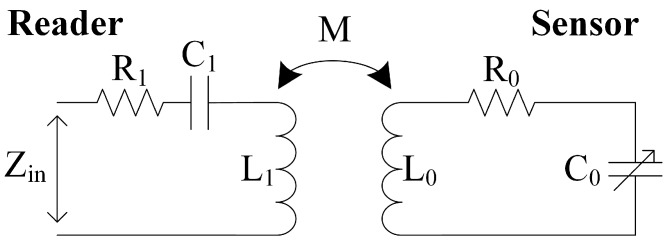
Equivalent circuit of the inductively-coupled sensor system.

Due to the mechanical stability of the LTCC material, LTCC-based sensors can be deployed in harsh environments. However, the signal readout and processing unit still need to be in a safe environment. One solution is the telemetry between the sensor and readout unit by inductive coupling, which has been applied to LTCC-based pressure sensors [19,20] and temperature sensors [21,22]. Wireless readout of the acceleration is first introduced to the field of LTCC accelerometers. The principle is shown in Figure 6. The acceleration signal is translated into resonance frequency changes by a variable capacitor, which is then detected through the coupling between the reader coil and the sensor coil.

Based on the circuit in Figure 6, the equivalent input impedance $Z_{in}$ at the reader coil port is given by:

(1) $$Z_{in}(j\omega )=R_{1}+j\omega L_{1}+\frac{1}{j\omega C_{1}}+\frac{\omega^{2}M^{2}}{R_{0}+j\omega L_{0}+\frac{1}{j\omega C_{0}}}$$

where $L_{1}$, $R_{1}$, and $C_{1}$ are the inductance, parasitic resistance, and parasitic capacitance of the reader coil, $L_{0}$ and $R_{0}$ are the inductance and parasitic resistance of the sensor coil. $C_{0}$ is the capacitance of the variable capacitor. M denotes the mutual inductance between reader coil and sensor coil, which is given by:

(2) $$M=k\sqrt{L_{1}L_{0}}$$

where k is the coupling coefficient with a value between 0 and ±1. When angular frequency ω equals $1/\sqrt{L_{0}C_{0}}$, which is the resonance frequency of the sensor circuit, the magnitude of $Z_{in}$ is at its maximum and, meanwhile, the phase of $Z_{in}$ will demonstrate a phase dip [23]. Thus, the resonance frequency of the sensor loop can be obtained by a frequency sweep on $Z_{in}$, and then picking its magnitude maximum.

Table 3 lists the physical dimensions of the passive components in the accelerometer. The square spiral inductor is used as the sensor’s coil, and its inductance can be derived with an empirical equation [24]:

(3) $$L=K_{1}\mu_{0}\frac{n^{2}d_{avg}}{1+K_{2}\rho }$$

where $K_{1}$ and $K_{2}$ are empirical coefficients dependent on the coil shape. For square coils, $K_{1}$ and $K_{2}$ are 2.34 and 2.75, respectively. $\mu_{0}$ is the permeability of a vacuum and n is the number of coil turns. The average diameter $d_{avg}$ is given by $d_{avg}=(d_{in}+d_{out})/2$, and the fill ratio ρ is given by $\rho =(d_{out}-d_{in})/(d_{out}+d_{in})$, where $d_{in}$ and $d_{out}$ are the inner and outer diameter, respectively. The calculated inductance is 1.26 μH and the initial capacitance of the variable capacitor is estimated as 12.744 pF by the plate capacitance formula. Therefore, the calculated resonance frequency is 39.72 MHz.

**Figure 7:**
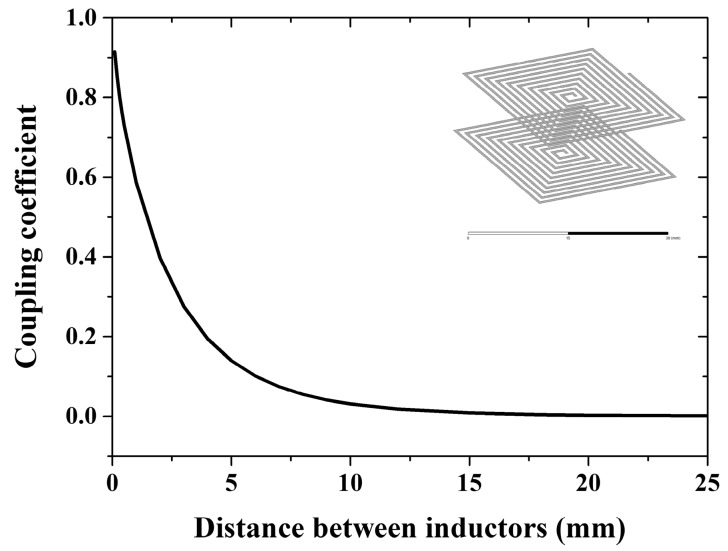
Effect of distance between inductors on the coupling coefficient.

As indicated by Equation (1), different mutual inductance M results in different maximal magnitude of $Z_{in}$ at resonance frequency. Increasing M is the most straightforward method to increase the signal-to-noise ratio of the system. Therefore, the sensor coil and read coil should be placed close enough to maintain a detectable impedance change. The impact of distance on coupling coefficient was investigated with the electromagnetic field solver ANSYS Q3D (ANSYS Inc., Canonsburg, PA, USA). The simulated inductors (both the sensor’s and reader’s) are of the same size with the one we used in the accelerometer. The results, as shown in Figure 7, demonstrate that, if the distance is larger than 5 mm, the coupling coefficient is too small to be detected (k < 0.1). If a longer distance is desired for a specific application, increasing the inductor diameter can solve the problem, but at the expense of small size.

### 3.2. Differential Capacitive Sensing

With the advantages of cancelling out the common-mode noise and high sensitivity, differential capacitive sensing is very common in accelerometers. The profile of the LTCC-based accelerometer utilizing differential capacitive sensing is shown in Figure 2b.

**Figure 8:**
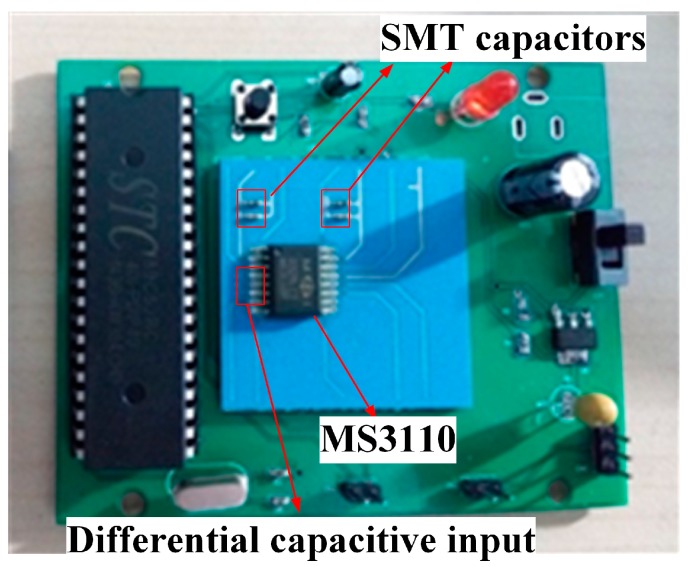
Differential capacitive accelerometer integrated with the signal processing circuits.

Figure 8 shows the evaluation board used for signal processing of the differential capacitive accelerometer. A commercially-available MS3110 (MicroSensors, Costa Mesa, CA, USA) readout chip was bonded onto the top cover. In addition, four 0306 SMT (surface mount technology) capacitors (Murata, Kyoto, Japan) were placed on the surface as filtering capacitors. Then, the differential capacitive input can be translated into the voltage output by the readout chip. From the datasheet of the MS3110, the transfer function between the differential capacitance and output voltage is given by:

(4) $$V_{out}=\frac{2.25\cdot 1.14\cdot Gain\cdot \Delta C}{CF}+VREF$$

where ΔC is the differential capacitance. The reference voltage VREF is 2.25 V, the feedback capacitor is 7.296 pF, and Gain is set to 4 in the experiment. These parameters can be set with the peripheral circuits on the evaluation board.

## 4. Performance Characterization

In this part, a static gravitational field test was performed for both the LC resonant accelerometer and the differential capacitive accelerometer to measure the acceleration sensitivity. The dynamic performance of the differential capacitive accelerometer was also evaluated using a vibration exciter. The results were obtained by averaging three repeated measurements for error reduction.

### 4.1. LC Resonant Accelerometer

**Figure 9:**
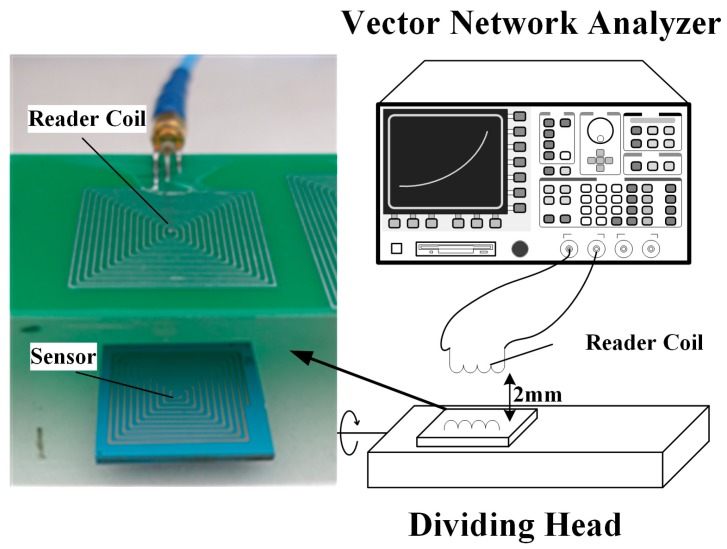
Dividing head test setup.

A static gravitational field test was carried out on the LC resonant accelerometer with a dividing head, which could be easily and precisely rotated to preset angles or circular divisions with an angle error of less than 1 s. Figure 9 shows the setup of the test environment. The accelerometer was stuck to the dividing head table. The reader coil is 2 mm above the sensor, and connected to an AV3629A vector network analyzer (VNA, CETI, Shandong, China). The VNA measured the 1-port S-parameter (scattering parameter of the reader coil, which is a 1-port network) from 0.1 to 100 MHz. The input impedance is then derived by:

(5) $$Z_{in}=Z_{0}\frac{1+S_{11}}{1-S_{11}}$$

where $S_{11}$ is the one-port S-parameter, and $Z_{0}$ is the reference impedance of the system, which is 50 Ω in this case.

**Figure 10:**
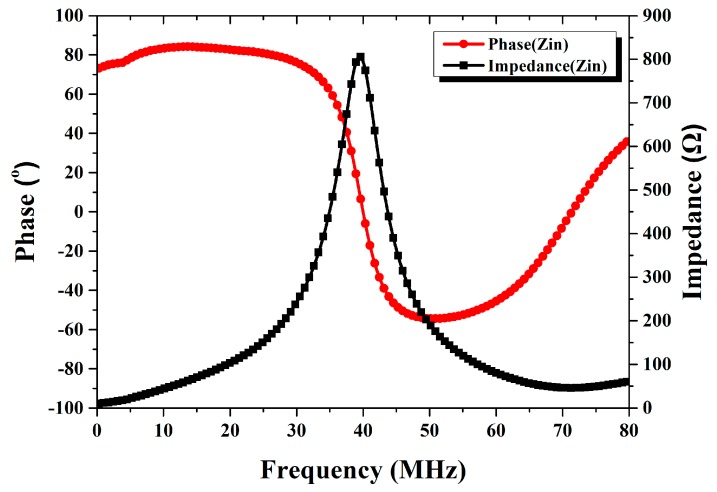
The impedance and phase of the input impedance with an acceleration of 1 g.

**Figure 11:**
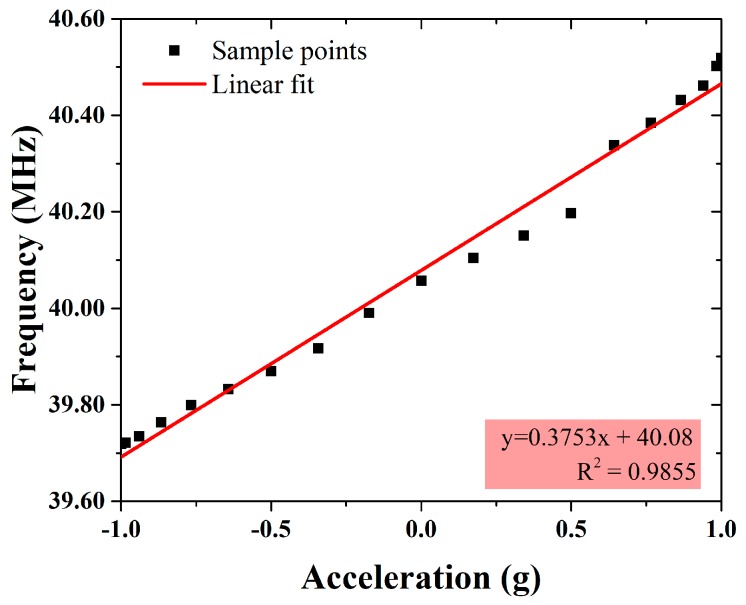
Resonance frequency vs. input acceleration.

The L-shaped beam described in Section 2 is used in the LC resonant accelerometer to guarantee a high sensitivity, in which case the calculate resonance frequency is 39.72 MHz and the estimated sensitivity is 598 KHz/g. The measured magnitude and phase of the input impedance with the acceleration of 1 g (the sensor inductor side faces up) is shown in Figure 10. The magnitude reaches its maximum value at 39.73 MHz, which is the resonance frequency of the sensor circuit. This corresponds well to the calculation. Figure 11 shows the measured resonance frequency vs. input acceleration using the dividing head (Tianhe Mechanical and Electrical Company, Shanghai, China). As the dividing head rotates from 0° to 180° (data was sampled once every 10°), the acceleration applied on the sensor changes from 1 g to −1 g, and the capacitance of the movable capacitor is increasing, which results in a decreasing resonance frequency. Zero offset is calculated as 40.12 MHz by averaging the outputs of accelerometer when acceleration is ±1 g. The measured sensor’s sensitivity is 375 KHz/g (equivalent to 1.88 μm/g in displacement), which is smaller than the estimate, may be caused by the slight distortion of the long-beam structure as shown in Figure 5b, because the fabricated beams are not as ideal as the ones in the simulation, and deformation occurs in the center area of beams and degrades performance. The nonlinearity is caused by the measurement error, because the long cable connecting the reader coil and VNA is very sensitive. Even with careful calibration, the parasitic effect induced by the cable can be changed by slight movement during the experiment. This can be solved by designing a compact signal processing circuit into the reader, which should include functionalities of frequency sweep, demodulation, and peak value extraction.

**Figure 12:**
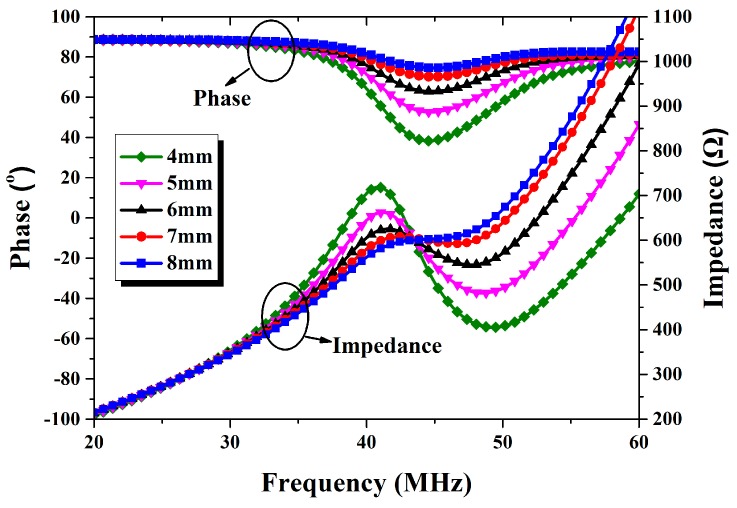
The impedance and phase of input impedance changes at different distances.

To determine the effective distance of wireless transmission, input impedance is measured at different distances of the reader coil and sensor, and the results are shown in Figure 12. As the distance increases from 4 to 8 mm, the V-shaped pattern formed by the phase curve becomes narrower and shallower, resulting in a poor signal-to-noise ratio. Similar results are also observed in impedance magnitude: the maximum value at the resonance frequency is decreasing, and the resonance wave is disappearing. The results indicate that the effective wireless transmission distance is 5 mm for our design.

### 4.2. Differential Capacitive Accelerometer

The performances of the differential capacitive accelerometer are reported in this part. The differential capacitive accelerometer uses Z-shaped beams. First of all, the zero offset was measured. By averaging the output voltage with dividing head at 0° and 180° (acceleration input is ±1 g), the zero offset in differential capacitance was calculated as 3.20 fF by Equation (4), with the output voltage being 2.255 V.

**Figure 13:**
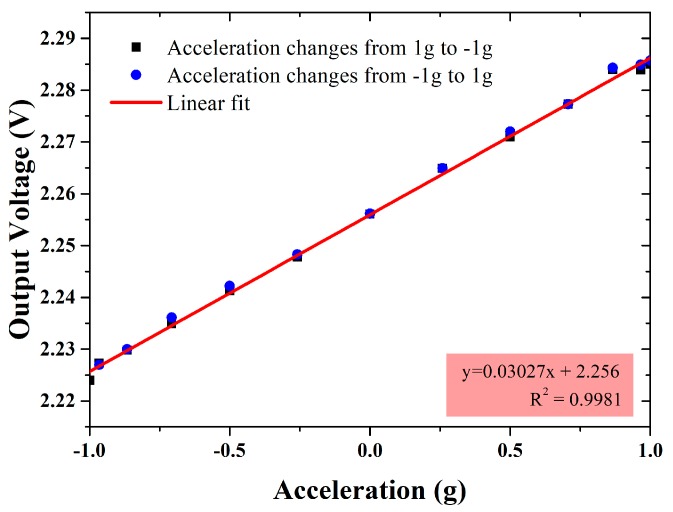
Dividing head test results of the differential capacitive accelerometer.

The dividing head test was also carried out for the differential capacitive accelerometer. The input acceleration is from 1 g to −1 g and then back to 1 g as the dividing head rotates from 0° to 360°, and the results is shown in Figure 13. The measured sensitivity in the dividing head test is 30.27 mV/g, equivalent to 21.50 fF/g in differential capacitance and 0.844 μm/g in displacement, and nonlinearity is less than 1%.

**Figure 14:**
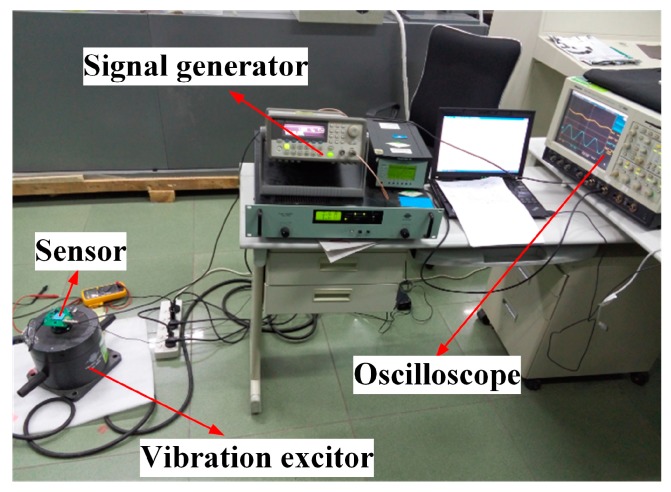
Test environment for the accelerometer dynamic performance.

**Figure 15:**
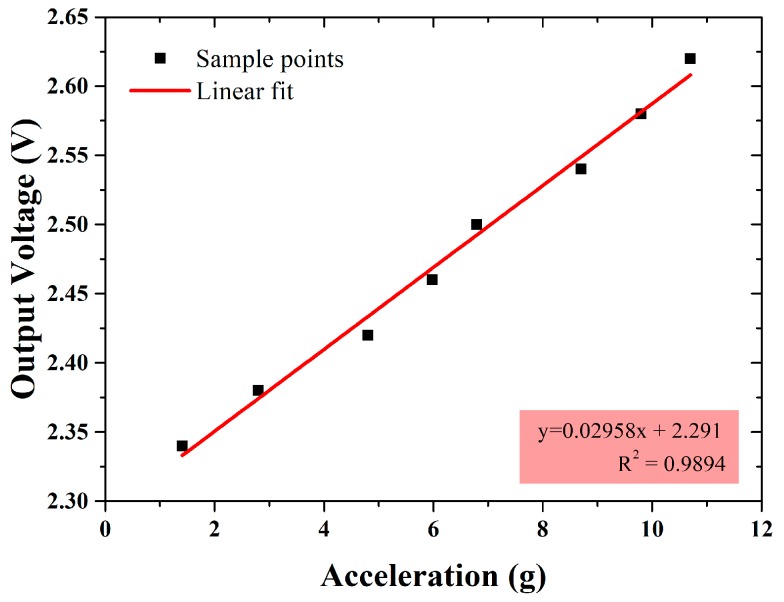
Vibration exciter test results of the differential capacitive accelerometer.

Subsequently, the dynamic performance of the accelerometer was characterized with a vibration exciter (Bruel and Kjaer, Copenhagen, Denmark). Figure 14 shows the test environment. The vibration frequency and amplitude were controlled by the signal generator (Agilent Technologies, Santa Clara, CA, USA) and power amplifier (SINOCERA, Shanghai, China). An 80 Hz sinusoidal acceleration input of different amplitude was used to drive the accelerometer. As the amplitude increases from 1.41 to 10.7 g, the sensor’s peak output voltage is increasing from 2.344 to 2.625 V, as shown in Figure 15. Therefore, the measured sensitivity in the vibration test is 29.58 mV/g with the nonlinearity less than 2%, and the full-scale range is over 10 g.

The performance of both the LC resonant accelerometer and the differential capacitive accelerometer is summarized in Table 4. It is noted that because the two accelerometers are based on different detection techniques, their sensitivity is in different units, and equivalent displacement sensitivity is provided in brackets for convenient comparison. Since the Z-shaped beams are stiffer than the L-shaped beams, the sensitivity of LC resonant accelerometer is 20 times higher than the differential capacitive accelerometer. However, the differential capacitive accelerometer has a larger full scale range and better characteristics with regard to nonlinearity, which benefits from the stable signal detection method. In addition, a qualitative judgment about the accuracy of the two detection techniques can be obtained. Differential capacitive sensing could cancel out the common-mode noise which still exists in coupling resonance frequency sensing. Additionally, wireless transmission without any shielding measures are more sensitive to electromagnetic noise in the environment than reliable wired interconnections, thus, differential capacitive sensing is more accurate compared with coupling resonance frequency sensing.

## 5. Conclusions

Two versions of LTCC-based capacitive accelerometers with different detection methods and mechanical structures are developed in this paper. The optimized LTCC process is effective in fabricating the key structures, such as the heavy proof mass with thin beams embedded in a large cavity. The LC resonant accelerometer has a high sensitivity 375 kHz/g over the full scale range of 1 g, and the separated sensor part and reader circuit facilitates the application of the accelerometer in harsh environments, with wireless readout achieved as far as 5 mm. The differential capacitive accelerometer demonstrates a stable performance of sensitivity of 30.27 mV/g, with nonlinearity less than 1% over the range ±1 g, and a full scale range over 10 g. This type of accelerometer can be used for navigation in dynamic vehicles. The future work is to reduce the size of the accelerometers by fabricating capacitor electrodes and the inductor coil distributed on different layers of LTCC tapes, and to realize three-axis inertial measurement.

## Figures and Tables

**Table 1:** Physical dimensions of the accelerometer.

Dimension Parameters	Value
Middle frame/top cover/bottom cover	30 mm × 30 mm × 1.5 mm/0.4 mm/0.4 mm
Cavity	22 mm × 22 mm × 1.7 mm
Proof mass	12 mm × 12 mm × 1.5 mm
L-shapedd beam	16 mm × 1 mm × 0.3 mm
Z-shapedd beam	6 mm × 1 mm × 0.3 mm
Anchor of Z-shapedd beam	3 mm × 3 mm × 0.3 mm
Capacitive gap	0.1 mm

**Table 2:** Simulated mechanical properties of beam-mass structure.

Parameters	L-Shaped Beams	Z-Shaped Beams
Displacement sensitivity	2.99 μm/g	0.321 μm/g
First resonance frequency	291 Hz	885 Hz
Second resonance frequency	634 Hz	1549 Hz
Third resonance frequency	634 Hz	1549 Hz

**Table 3:** Physical dimensions of the inductor and capacitor.

Parameters	Value
Inner diameter of inductor coil	2.5 mm
Outer diameter of inductor coil	25.3 mm
Number of turns	10
Line width of coil	0.5 mm
Line spacing of coil	0.7 mm
Capacitor dimension	12 mm × 12 mm
Gap between capacitor electrodes	0.1 mm

**Table 4:** Comparison of the LC resonant accelerometer and differential capacitive accelerometer.

Parameters	LC Resonant Accelerometer	Differential Capacitive Accelerometer
Type of beams	L-shaped beams	Z-shaped beams
Sensitivity	375 kHz/g (1.88 μm/g)	30.27 mV/g (0.844 μm/g)
Full scale range	1 g	10 g
Zero offset	40.12 MHz	2.255 V
Nonlinearity	Less than 6%	Less than 1%
